# USP9X stabilizes BRCA1 and confers resistance to DNA‐damaging agents in human cancer cells

**DOI:** 10.1002/cam4.2528

**Published:** 2019-09-11

**Authors:** Qin Lu, Fang‐Lin Zhang, Da‐Yun Lu, Zhi‐Ming Shao, Da-Qiang Li

**Affiliations:** ^1^ Shanghai Cancer Center and Institutes of Biomedical Sciences Shanghai Medical College Fudan University Shanghai China; ^2^ Cancer Institute Shanghai Medical College Fudan University Shanghai China; ^3^ Department of Oncology Shanghai Medical College Fudan University Shanghai China; ^4^ Department of Analytical Chemistry and CAS Key Laboratory of Receptor Research Shanghai Institute of Materia Medica Chinese Academy of Sciences Shanghai China; ^5^ Department of Breast Surgery Shanghai Medical College Fudan University Shanghai China; ^6^ Key Laboratory of Breast Cancer in Shanghai Shanghai Medical College Fudan University Shanghai China

**Keywords:** BRCA1, breast cancer, deubiquitinase, PARP inhibitor, USP9X

## Abstract

BRCA1, a multifunctional protein with an important role in DNA double‐strand break repair by homologous recombination (HR), is subjected to ubiquitin‐dependent degradation. To date, several E3 ubiquitin ligases have been identified to govern BRCA1 stability, but the deubiquitinase that counteracts its turnover remains undefined. In this study, we report that the ubiquitin‐specific protease 9X (USP9X) is a *bona fide* deubiquitinase for BRCA1 in human cancer cells. Reciprocal immunoprecipitation assays demonstrated that USP9X interacted with BRCA1. Depletion of USP9X by short interfering RNAs or inhibition of USP9X by the small‐molecular inhibitor WP1130 significantly reduced BRCA1 protein abundance, without affecting its mRNA levels. In contrast, overexpression of wild‐type USP9X, but not its deubiquitinase activity‐defective mutant (C1566S), resulted in an upregulation of BRCA1 protein levels. Moreover, USP9X depletion reduced the half‐life of BRCA1, accompanied by an increase in its ubiquitination. HR assays showed that knockdown of USP9X significantly reduced HR efficiency, which was partially rescued by reintroduction of BRCA1 into USP9X‐depleted cells. In support of these findings, USP9X knockdown significantly enhanced sensitivity to PARP inhibitor Olaparib and methyl methanesulfonate (MMS). Collectively, these results establish USP9X as a deubiquitinase for BRCA1 and reveal a previously unrecognized role of USP9X in the regulation of HR repair and the sensitivity of cancer cells to DNA‐damaging agents.

## Graphical abstract

In this study, we identified USP9X as the first deubiquitinase of BRCA1. Consequently, USP9X regulates BRCA1‐mediated HR repair and confers resistance to DNA‐damaging agents in human cancer cells.

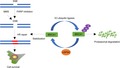

## 1 INTRODUCTION

The BRCA1 tumor suppressor is a multifunctional nuclear protein participating in a multitude of fundamental cellular processes, especially DNA damage response (DDR).^1^, ^2^ During DDR, BRCA1 forms various complexes by interacting with different partners, including CtIP,^3^ CCDC98,^4^, ^5^ and BACH1.^6^, ^7^ These complexes are recruited to sites of DNA lesions and facilitate efficient repair of DNA double‐stranded breaks (DSBs) through homologous recombination (HR).^8^ Consequently, loss or mutation of BRCA1 leads to genomic instability and tumorigenesis.^9^ Cells with BRCA1 deficiency or mutations have enhanced sensitivity to poly (ADP‐ribose) polymerase (PARP) inhibitors and DNA‐damaging chemotherapeutic agents.^10^, ^11^, ^12^ Thus, unraveling the regulatory mechanisms of BRCA1 in human cancer cells would promote the advances in the prevention and treatment of human cancers.

Emerging evidence shows that the ubiquitin‐proteasomal system is involved in the regulation of BRCA1 stability.^13^, ^14^ In this context, the ubiquitin‐conjugating enzyme E2T (UBE2T),^15^ the HECT family of E3 ubiquitin ligases, HERC2 (HECT and RLD domain containing E3 ubiquitin protein ligase 2)^16^ and HUWE1 (HECT, UBA and WWE domain containing E3 ubiquitin protein ligase 1),^17^ and the F‐box protein 44 (FBXO44),^18^ a component of the SCF (SKP1‐CUL1‐F‐box protein)‐type E3 ubiquitin ligase complex, have been shown to mediate BRCA1 ubiquitination and subsequent proteasomal degradation.^16^, ^17^ Moreover, tumor suppressor candidate 4 (TUSC4) can block the binding of HERC2 to BRCA1, thereby suppressing BRCA1 ubiquitination and proteasomal degradation.^19^ Cathepsin S, a cysteine protease, regulates ubiquitin‐mediated degradation of BRCA1 and suppresses BRCA1‐mediated HR repair activity.^20^ Despite these advances, the deubiquitinating enzymes (DUBs) that counteract BRCA1 ubiquitination and degradation have not been identified to date.

Ubiquitin‐specific peptidase 9X (USP9X) is a highly conserved DUB belonging to the ubiquitin‐specific protease (USP) family.^21^ Accumulating evidence shows that USP9X is frequently upregulated and promotes tumorigenesis and chemoresistance in some types of human cancer, such as breast^22^, ^23^, ^24^ and lung cancer,^25^, ^26^ melanoma,^27^ lymphoma,^28^, ^29^ and glioblastoma.^30^ Strikingly, a tumor suppressor role of USP9X has been documented in pancreatic,^31^, ^32^, ^33^ colorectal,^34^ and renal cancer.^35^ The complex role of USP9X in human cancers is determined by its various substrates. Recently, a high‐throughput quantitative proteomic analysis to identify the potential substrates of USP9X using wild‐type (WT) and USP9X‐depleted HeLa cells indicates that BRCA1 could be regulated by USP9X.^36^ Considering the functional importance of BRCA1 in human cancer development and therapeutic responsiveness, in this study we aimed to address the functional and mechanistic role of USP9X in the regulation of BRCA1 in human cancer cells.

Here, we provide evidence that USP9X stabilizes BRCA1 by antagonizing its ubiquitination. Functional experiments further demonstrated that the USP9X‐BRCA1 signaling axis is involved in regulating HR repair and, consequently, the sensitivity of cancer cells to DNA‐damaging agents.

## 2 MATERIALS AND METHODS

### 2.1 Cell culture and chemical reagents

Human cervical adenocarcinoma cell line HeLa, human embryonic kidney epithelial cell line HEK293T, human breast cancer cell lines MCF‐7, T47D, MDA‐MB‐231, and BT549 were obtained from the Type Culture Collection of the Chinese Academy of Sciences. These cell lines were authenticated by short tandem repeat profiling and were mycoplasma‐free. All cell lines were cultured in Dulbecco's modified Eagle's medium (BasalMedia) supplemented with 10% fetal bovine serum (ExcellBio) and 1% penicillin/streptomycin (BasalMedia). The protein synthesis inhibitor cycloheximide (CHX) and the DNA‐damaging agent methyl methanesulfonate (MMS) were purchased from Cell Signaling Technology and Sigma‐Aldrich, respectively. Proteasome inhibitor MG‐132 and PARP inhibitor Olaparib were from Selleck.

### 2.2 Expression vectors

The pEF‐DEST51 empty vector and plasmids encoding pEF‐DEST51‐V5‐USP9X and pEF‐DEST51‐V5‐USP9X C1566S (catalytically inactive mutant) were kindly provided by Dr Stephen A. Wood (Eskitis Institute for Cell and Molecular Therapies, Griffith University) and have been described previously.^37^, ^38^ Myc‐DDK‐tagged BRCA1 cDNA was purchased from Origene. The pDR‐GFP and ISceI‐GR expression vectors were from Addgene and YouBio, respectively. Hemagglutinin (HA)‐tagged ubiquitin (HA‐ubiquitin), small hairpin RNA (shRNA) targeting USP9X (shUSP9X), and negative control shRNA (shNC) were kindly provided by Dr Hu Zhou (Shanghai Institute of *Materia Medica*, Chinese Academy of Sciences). Small interfering RNA (siRNA) targeting USP9X (siUSP9X) and negative control siRNA (siNC) were synthesized by GenePharma. The shRNA and siRNA targeting sequences are provided in Table S1–S3.

### 2.3 Plasmid and siRNA transfection

Cells were seeded onto 6‐well plates or 10‐mm dishes and plasmid transfection was performed using Lipofectamine 2000 (Invitrogen) or Neofect DNA transfection reagent (TengyiBio) when cell confluency was about 70%. Transfection of siRNAs was carried out using Lipofectamine 2000 according to the manufacturer's protocol. Briefly, cells were seeded onto 6‐well plates and transfected with siRNAs when cell confluency was about 50%. After 48 hours of transfection, cells were harvested for immunoblotting and quantitative Real‐Time PCR (qRT‐PCR) analysis, respectively.

### 2.4 Generation of USP9X knock down stable cell lines

HEK293T cells were cotransfected with shUSP9X lentiviral vectors and packaging plasmids psPAX2 and pMD2.G using Neofect DNA transfection reagent. After 36‐48 hours of transfection, the viral supernatant was collected, centrifuged, and filtered through a 0.45‐μm filter. To generate stable cell lines expressing shUSP9X, cells were infected with the viral supernatant in complete medium supplemented with 10 μg/mL polybrene (Sigma‐Aldrich). Two days post infection, cells were cultured in complete medium in the presence of 1‐2 μg/mL puromycin (Cayman) for another 2 weeks. Stable USP9X‐knockdown cells were maintained in complete medium supplemented with 1‐2 μg/mL puromycin.

### 2.5 RNA extraction and qRT‐PCR

Total RNA was isolated with TRIzol Reagent (Invitrogen) according to the manufacturer's protocol. RNA pellet was resuspended in 30 μL RNase‐free water, and RNA yield was determined using NanoDrop spectophotometer (Thermofisher)**.** Then, equal amounts of RNA were converted to cDNAs using PrimeScript^TM^ Reverse Transcription Master Mix (Takara). qRT‐PCR was performed using SYBR Premix Ex Taq (Tli RNaseH Plus, Takara). GAPDH was used as an internal control. The primer information of USP9X, BRCA1 and GAPDH is provided in Table S1–S3.

### 2.6 Antibodies, immunoblotting, immunoprecipitation assays, and immunofluorescent staining

Primary antibodies used in this study are listed in Table S1–S3. The HRP‐linked secondary antibodies were purchased from Cell Signaling Technology. Immunoblotting analysis, immunoprecipitation assays, and immunofluorescent staining were performed as described previously in details.^39^, ^40^, ^41^

### 2.7 In vivo ubiquitination assay

In vivo ubiquitination assay was performed as described previously.^39^, ^40^ Briefly, HEK293T cells were seeded into 10‐cm dish overnight. Transfection of siRNAs targeting USP9X (siUSP9X) or siNC was performed using Lipofectamine 2000. After 6 hours, cells were cotransfected with HA‐ubiquitin and Flag‐BRCA1 using Neofect^TM^ DNA transfection reagent (TengyiBio). Two days post transfection, cells were treated with 10 μmol/L MG‐132 for 6 hours, and cell lysates were prepared and subjected to in vivo ubiquitination assays.

### 2.8 HR assays

HR assays were performed following the protocol described previously.^42^ Briefly, a clone stably expressing pDR‐GFP was generated and validated by analyzing GFP‐positive cells. The pDR‐GFP expressing cells were transfected with ISceI‐GR plasmids and treated with triamcinolone acetonide for 48 hours.^43^ The isolated clones that have 4% GFP‐positive cells were selected for subsequent analysis. For HR assays, cells stably expressing DR‐GFP and ISceI‐GR were transfected with siNC or siUSP9X, with or without BRCA1 expression vectors. Twenty four hours post transfection, cells were treated with 10 μmol/L triamcinolone acetonide and cultured for another 48 hours. The proportion of GFP‐positive cells was evaluated using flow cytometry and the efficiency of HR was calculated.

### 2.9 Colony formation and cell survival assays

For colony formation assay, cells were seeded onto a 12‐well plate (500 single cells per well) overnight. Cells were treated with dimethyl sulfoxide (DMSO) or Olaparib at the indicated doses and then the medium was replaced every 3 days. After 2 weeks, the cells were fixed with methanol for 30 minutes and then stained with 0.5% crystal violet stain solution for 1 hour. The colonies were imaged and the colony numbers were counted. For cell survival assays, cells were seeded into 96‐well plates (5 × 10^3^ cells per well), allowed to adhere overnight, and then treated with DMSO or MMS at indicated concentrations for 24 hours. Then, cells were cultured in fresh culture without MMS for another 24 hours. Cell viability was determined using Cell Counting Kit‐8 (CCK‐8) (Dojindo, Shanghai, China) according to the manufacturer's instructions.

### 2.10 Statistical analysis

For each experiment, at least three independent experiments were performed. Data from independent experiments were calculated and expressed as mean ± SD. Statistical analysis was carried out using a two‐tailed unpaired Student's *t* test, and *P* < .05 was considered statistically significant.

## 3 RESULTS

### 3.1 USP9X regulates BRCA1 expression at protein level

**Figure 1:**
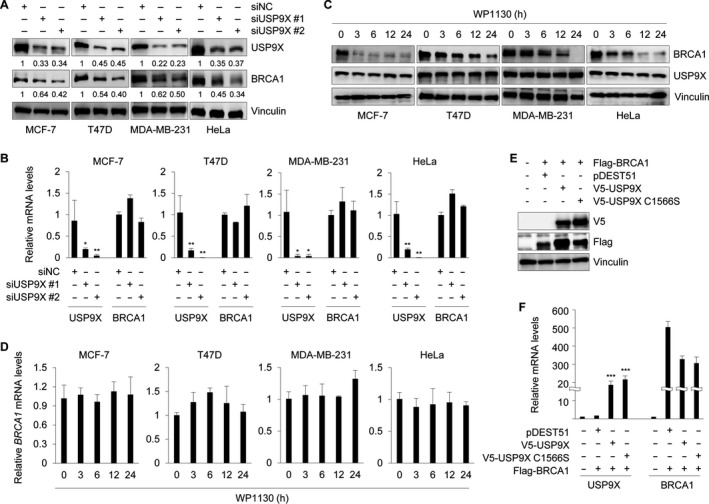
USP9X regulates BRCA1 at protein level. A and B, MCF‐7, T47D, MDA‐MB‐231, and HeLa cells were transfected with indicated siRNAs for 48 h. Cell lysates were subjected to Western blot analysis with the indicated antibodies (A) or qRT‐PCR (B). C and D, Cells were treated with or without 5 μmol/L WP1130 for indicated times. Cell lysates were subjected to immunoblotting (C) or qRT‐PCR (D) analysis. E and F, HEK293T cells were cotransfected with indicated expression vectors for 48 h. The protein and mRNA levels of USP9X and BRCA1 were evaluated using Western Blot and qRT‐PCR analysis, respectively. In B and F, *P < .05, **P < .01, ***P < .001

To test whether BRCA1 expression is regulated by USP9X, endogenous USP9X was depleted using two independent siUSP9Xs in three breast cancer cell lines (MCF‐7, T47D, and MDA‐MB‐231) and HeLa cells, which express wild‐type BRCA1.^44^, ^45^ Then, mRNA and protein levels of USP9X and BRCA1 were examined using immunoblotting and qRT‐PCR analysis, respectively. Results showed that USP9X depletion significantly reduced BRCA1 protein levels but did not affect its mRNA levels (Figure 1A,B). Similarly, inhibition of USP9X by a partially selective inhibitor WP1130^46^ reduced BRCA1 protein levels, but did not affect BRCA1 mRNA levels (Figure 1C,D). In contrast, overexpression of wild‐type USP9X, but not its catalytically inactive mutant (C1566S), upregulated the protein levels of exogenously expressed BRCA1 (Figure 1E). qRT‐PCR analysis showed that both wild‐type (WT) and catalytically inactive mutant USP9X did not increase but slightly decreased BRCA1 mRNA levels (Figure 1F). As both WT and the catalytically inactive mutant USP9X have similar inhibitory effects on BRCA1 mRNA levels, we speculated that USP9X may regulate the expression of some BRCA1 transcription‐related factors through a noncanonical, deubiquitination‐independent mechanism. For instance, the deubiquitinase ubiquitin‐specific protease 4 (USP4) has been shown to suppress MyoD activity in a catalytic activity independent manner.^47^ These results indicate the regulation of BRCA1 by USP9X to be posttranscriptional.

### 3.2 USP9X enhances the stability of BRCA1 and counteracts its ubiquitination

**Figure 2:**
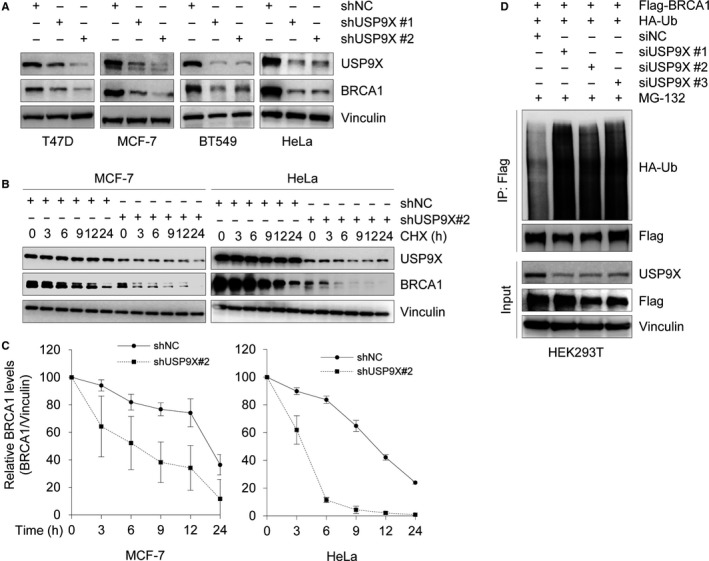
USP9X knockdown reduces BRCA1 stability and enhances its ubiquitination. A, Lysates from cells stably expressing shNC, shUSP9X#1 and shUSP9X#2 were subjected to immunoblotting analysis with the indicated antibodies. B and C, MCF‐7 and HeLa cells stably expressing shNC or shUSP9X were treated with 200 μg/mL cycloheximide (CHX) for the indicated times. Total cellular lysates were subjected to immunoblotting analysis with the indicated antibodies (B). Quantitative results of relative BRCA1 protein levels (BRCA1/Vinculin) from three independent experiments are shown in C. D, HEK293T cells were cotransfected with Flag‐BRCA1, HA‐ubiquitin (Ub), siNC, or siUSP9Xs (#1‐3) for 48 h. Then, cells were treated with 20 μmol/L MG‐132 for 6 h and then subjected to immunoprecipitation assays using Flag M2 affinity gel, followed by immunoblotting analysis with the indicated antibodies

In support of the above results, depletion of USP9X in T47D, MCF‐7, BT549, and HeLa cells by two independent USP9X shRNAs (shUSP9X #1 and #2) also significantly decreased BRCA1 protein levels (Figure 2A). Moreover, it was noticed that shUSP9X #2 knocked down USP9X more efficiently than shUSP9X #1. To test whether USP9X regulates BRCA1 protein stability, MCF‐7 and HeLa cells stably expressing shNC or shUSP9X #2 were treated with 200 μg/mL CHX. Samples were collected at the indicated times and then subjected to immunoblotting analysis with the indicated antibodies. As shown in Figure 2B,C, the half‐life of BRCA1 in cells expressing shUSP9X #2 was significantly shorter than that in cells expressing shNC, indicating that USP9X enhances the stability of BRCA1 protein. As USP9X is a substrate‐specific deubiquitinase,^21^ we next examined the effect of USP9X knockdown on BRCA1 ubiquitination. Toward this aim, HEK293T cells were transfected with Flag‐BRCA1, HA‐ubiquitin, siNC, or siUSP9X. After 48 hours of transfection, cells were treated with 10 μmol/L MG‐132 for 6 hours and then total cellular lysates were subjected to IP assays with Flag M2 affinity gel. Immunoblotting analysis showed that USP9X knockdown significantly increased the ubiquitination of BRCA1 protein (Figure 2D).

### 3.3 USP9X interacts with BRCA1

**Figure 3:**
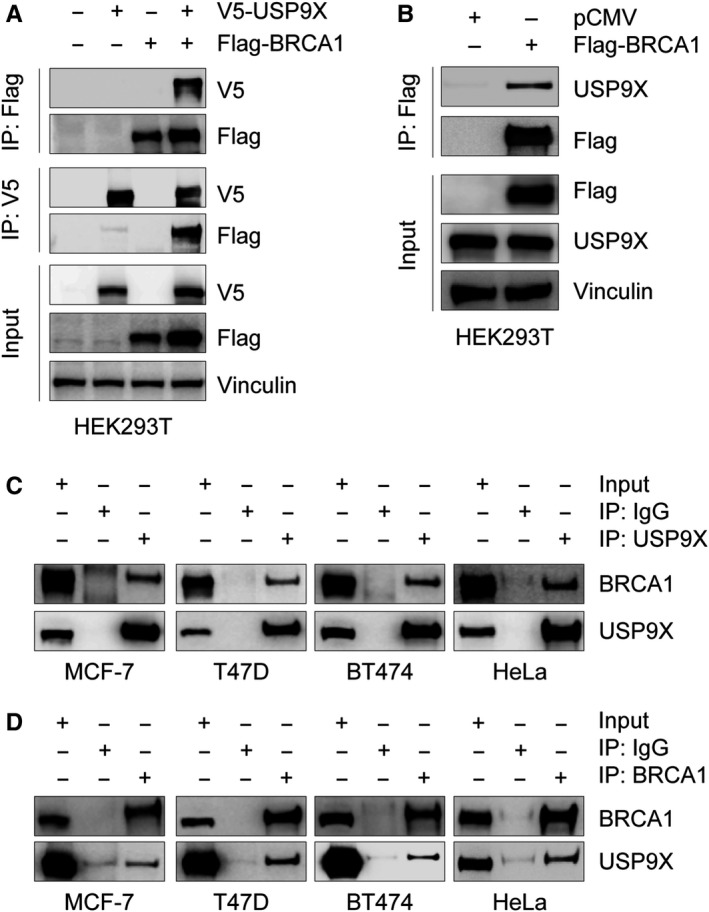
USP9X interacts with BRCA1. A and B, HEK293T cells were transfected with the indicated expression vectors. After 48 h of transfection, total cellular lysates were subjected to co‐immunoprecipitation and immunoblotting analysis with the indicated antibodies. C and D, Lysates from MCF‐7, T47D, BT474 or HeLa cells were immunoprecipitated with control IgG, anti‐USP9X (C) or anti‐BRCA1 (D) antibody, followed by immunoblotting analysis with the indicated antibodies

To address the mechanisms for USP9X regulation of BRCA1 stability, we next examined whether USP9X interacts with BRCA1. To do this, HEK293T cells were transfected with expression vectors encoding Flag‐BRCA1 and V5‐USP9X alone or in combination. After 48 hours of transfection, cells were harvested and were subjected to reciprocal co‐immunoprecipitation assays using either an anti‐Flag or an anti‐V5 antibody. As shown in Figure 3A, Flag‐BRCA1 was immunoprecipitated with V5‐USP9X when coexpressed. In addition, Flag‐BRCA1 was immunoprecipitated with endogenous USP9X in HEK293T cells (Figure 3B). To further determine the endogenous interaction between BRCA1 and USP9X, MCF‐7, T47D, BT474, and HeLa cells were subjected to reciprocal immunoprecipitation assays using either an anti‐BRCA1 or an anti‐USP9X antibody. As shown in Figure 3C,D, BRCA1 was immunoprecipitated with USP9X in those cell lines. Together, these results suggest that USP9X interacts with BRCA1.

### 3.4 USP9X promotes HR repair partially through BRCA1

**Figure 4:**
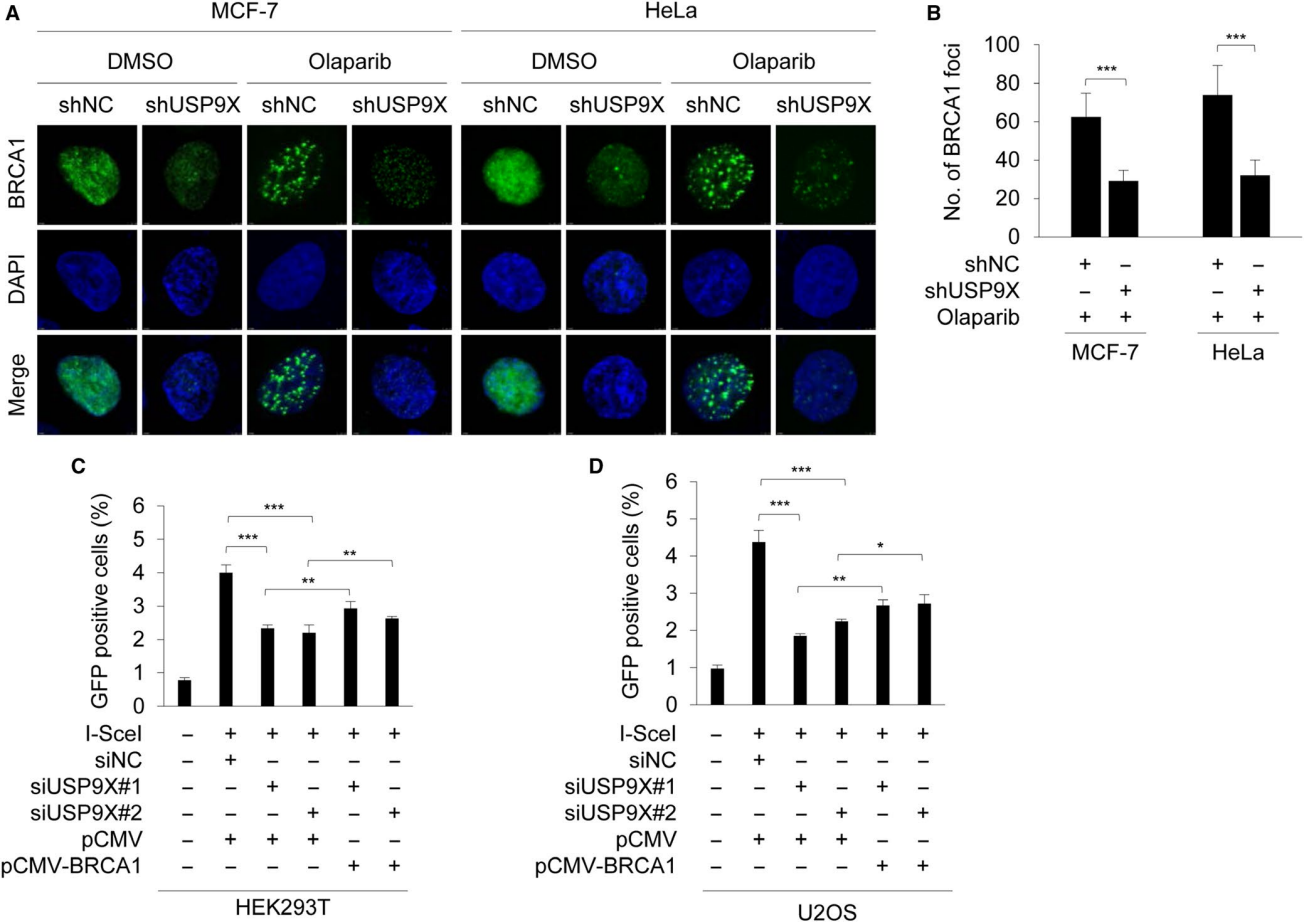
USP9X promotes HR repair through BRCA1. A and B, MCF‐7 and HeLa cells stably expressing shNC and shUSP9X #2 were treated with or without 20 μM Olaparib for 48 h and the BRCA1 foci formation was detected using immunofluorescence staining with an anti‐BRCA1 antibody. Representative immunofluorescence images and quantitative results of BRCA1 foci are shown in A and B, respectively. C and D, HEK293T (A) and U2OS (B) cells stably expressing DR‐GFP were cotransfected with the indicated siRNAs and expression vectors. After 24 h of transfection, cells were treated with 10 μmol/L triamcinolone acetonide for 48 h. GFP‐positive cells were analyzed using fluorescene‐activated cell sorting (FACS). Quantitative results of GFP‐positive cells are shown. In B, C, and D, *P < .5, **P < .01, ***P < .001

As it has been shown that treatment with PARP inhibitor Olaparib enables to induce BRCA1 foci formation in human cancer cells,^48^ we next examined whether depletion of USP9X could affect Olaparib induced the formation of BRCA1 foci using immunofluorescent staining. Results showed that treatment with Olaparib markedly induced BRCA1 foci formation in shNC expressing cells, but the noted effects were compromised upon USP9X depletion (Figure 4A,B). To determine the involvement of the USP9X‐BRCA1 signaling axis in HR repair, we developed a cell system in which DSB at a defined genomic site can be induced by expression of ISceI endonuclease. The efficiency of HR was evaluated using FACS analysis of GFP‐positive cells.^42^, ^43^ As expected, cells stably expressing DR‐GFP substrate alone had no detectable GFP. In contrast, approximately 4% cells expressing ISceI endonuclease were GFP positive. Depletion of USP9X by siRNAs significantly reduced the proportion of GFP‐positive cells, and this effect was partially rescued by reintroduction of BRCA1 into cells with USP9X knockdown (Figure 4C,D). Together, these data suggest that USP9X promotes HR repair of DSB partially through BRCA1.

### 3.5 USP9X depletion increases the sensitivity of cancer cells to DNA‐damaging agents

**Figure 5:**
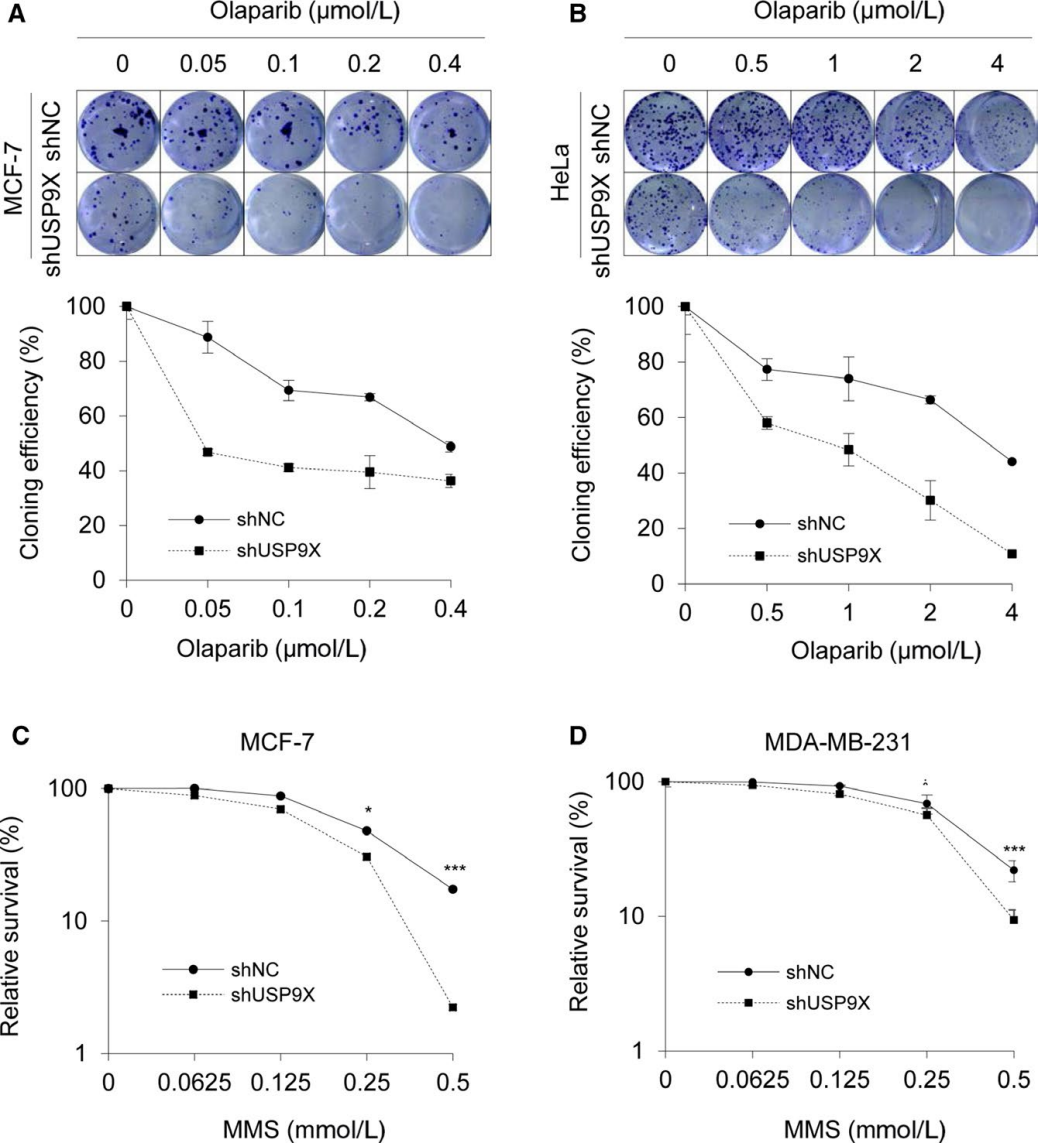
Depletion of USP9X results in enhanced cellular sensitivity to DNA‐damaging agents. A and B, MCF‐7 and HeLa cells stably expressing shNC and shUSP9X were treated with or without Olaparib at the indicated concentrations and incubated until colonies were formed. Colonies were stained with crystal violet solution (upper panel). The cloning efficiency (lower panel) was calculated by normalizing each group to vehicle treatment group. (C‐D) MCF‐7 and MDA‐MB‐231 cells stably expressing shNC and shUSP9X #2 were treated with the indicated concentrations of MMS for 24 h. Cells were incubated in fresh medium without MMS for another 24 h, and cell survival was assayed using CCK‐8 kit. In C and D, *P < .05, **P < .01, ***P < .001

In the setting of decreased HR activity induced by BRCA1 deficient, PARP inhibition leads to chromatid aberrations and cell lethality.^49^ To evaluate whether HR impairment following USP9X depletion increases sensitivity to PARP inhibition, we carried out colony formation assays using MCF‐7 and HeLa cells stably expressing shNC and shUSP9X in the presence or absence of PARP inhibitor Olaparib at indicated doses. Results showed that knockdown of USP9X by shRNAs increased the sensitivity of MCF‐7 and HeLa cells to Olaparib (Figure 5A,B).

In addition, it has been shown that HR defective cells are sensitive to MMS‐induced DNA damage.^11^, ^50^ Next, we determined the effect of USP9X depletion on cell viability after treatment with MMS using CCK‐8 assays. As shown in Figure 5C,D, depletion of USP9X in MCF‐7 and MDA‐MB‐231 cells enhanced the cellular sensitivity to MMS. Collectively, these results suggest that USP9X regulates BRCA1 stability and cellular sensitivity to DNA‐damaging agents.

## 4 DISCUSSION

**Figure 6:**
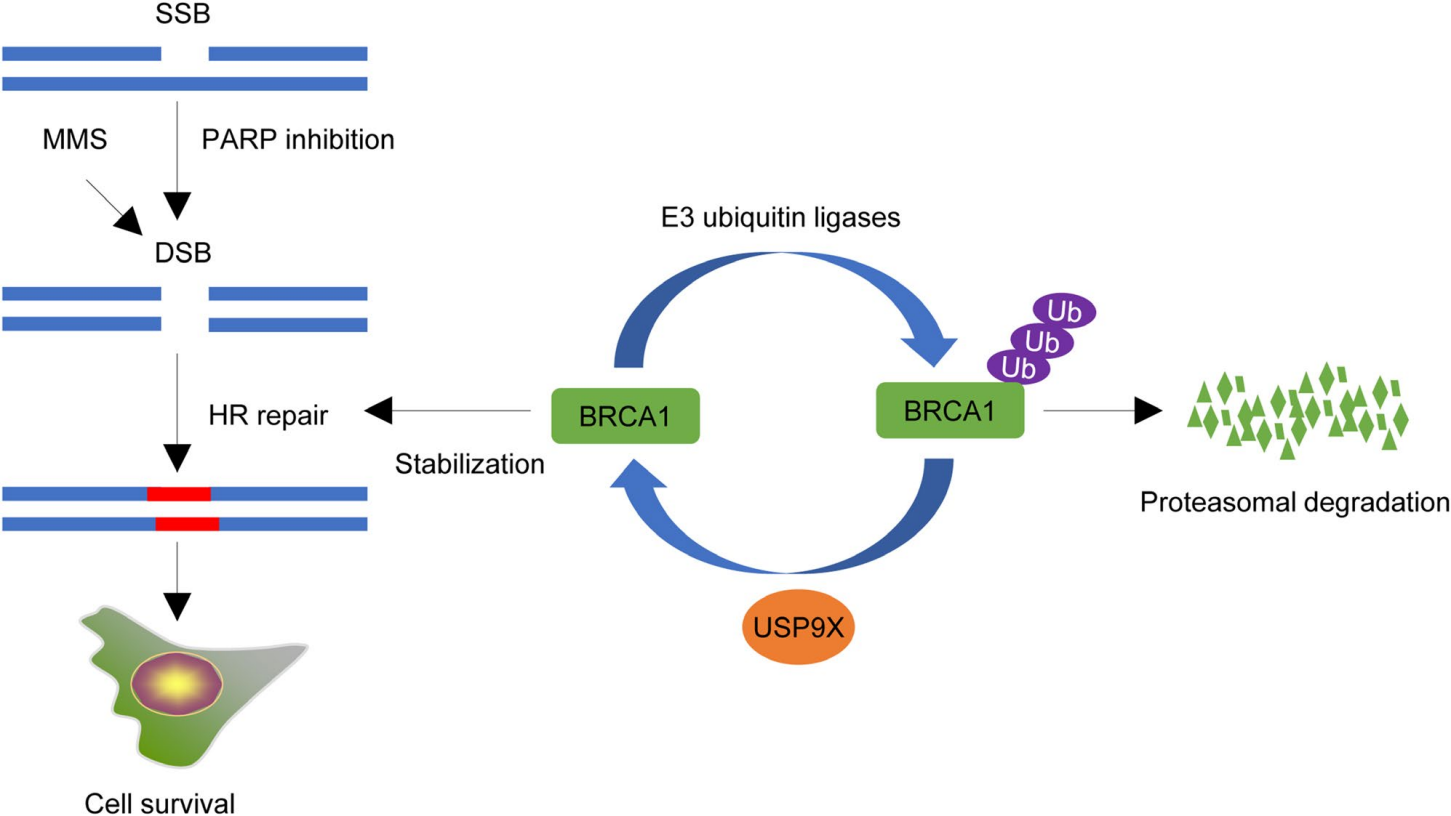
The proposed working model. USP9X deubiquitinates and stabilizes BRCA1, which in turn promotes HR repair of MMS‐ and PARP inhibitor Olapairb‐induced DSBs and cell survival

Ubiquitination is a fundamental mechanism for regulating protein turnover and stability, which is dynamically regulated by ubiqitinating enzymes and deubiquitinating enzymes (DUBs).^51^ The human genome encodes almost 100 deubiquitylating enzymes (DUBs),^52^ and some of them, such as USP7,^53^ USP11,^54^ USP15,^55^ USP21,^56^ USP34,^57^ USP47,^58^ USP51,^59^ have been shown to be involved in regulating DNA repair and maintaining genome integrity. In the present study, we found that USP9X functions as a deubiquitinase of BRCA1. Moreover, USP9X regulates BRCA1‐mediated HR repair and promotes resistance of cancer cells to DNA‐damaging agents (Figure 6).

BRCA1 functions as a tumor suppressor, which is essential for the maintenance of genome integrity and suppression of malignant neoplasms.^60^ Although several E3 ubiqitinating enzymes have been documented to regulate BRCA1 ubiquitination and degradation,^15^, ^16^, ^17^, ^18^ no specific DUBs are known to control BRCA1 protein stability. In this study, we found that knockdown or inhibition of USP9X remarkably decreases BRCA1 protein but not mRNA levels (Figure 1). USP9X is a highly conserved deubiquitinating enzyme belonging to the USP family.^21^ The USP family members share a catalytic domain, which contains two short conserved cysteine and histidine catalytic motifs.^21^, ^52^, ^61^ Therefore, the cysteine and histidine catalytic motifs in the catalytic domain of USP9X are responsible for its deubiquitinase activity. Consistent with this, several previous studies have reported that USP9X mutant C1599A,^62^ C1566A,^63^ C1566S,^25^ and H1871A^31^ could reduce its deubiquitination activity. In our experiments, overexpression of wild‐type, but not C1566S mutant, USP9X significantly affects BRCA1 abundance (Figure 1E), suggesting that USP9X regulation of BRCA1 protein levels depends on its deubiquitinase activity. Moreover, USP9X interacts with BRCA1 (Figure 3A,D), and USP9X silencing by siRNAs resulted in an increase of BRCA1 ubiquitination (Figure 2D). These results indicate that USP9X is a novel stabilizer for BRCA1 by antagonizing its ubiquitination.

BRCA1 participates in various DNA repair signaling pathways, in particular, in DSB repair by HR.^64^, ^65^ Considering the results that USP9X depletion significantly reduced the stability of BRCA1 (Figure 2), we proposed that USP9X may be implicated in DSB repair. As expected, the results from fluorescence‐based assays demonstrated that siRNA‐mediated USP9X knockdown remarkably hindered the efficiency of HR‐mediated DSB repair, while introduction of BRCA1 in USP9X‐‐depleted cells partially rescued this effect (Figure 4). Consistently, USP9X depletion enhanced cellular sensitivity to PARP inhibitor Olaparib and DNA‐damaging agent MMS (Figure 5). In support of our findings, the deubiquitinase USP13 has been shown to deubiquitinate BRCA1‐interacting protein RAP80 and to promote proper DDR.^66^ Consequently, overexpression of USP13 renders ovarian cancer cells resistant to chemotherapeutic drug cisplatin and PARP inhibitor Olaparib.^66^ Similarly, USP15 regulates HR repair by deubiquitinating BARD1, a major BRCA1 binding partner, and decreases PARP inhibitor sensitivity in cancer cells.^55^ USP21 deubiquitinates and stabilizes BRCA2 in hepatocellular carcinoma cells to promote tumor cell growth.^56^ USP7 deubiquitinates and stabilizes MDC1, an essential player in the sensing and repair of DSBs, to regulate DDR.^53^ Moreover, USP7 contributes to cervical carcinogenesis and its expression levels are associated with worse survival rates for patients with cervical cancer.^53^

In summary, the findings presented here suggest that USP9X is a novel binding partner of BRCA1 and stabilizes BRCA1. Moreover, knockdown of USP9X enhances the sensitivity of human cancer cells to PARP inhibitor Olaparib and MMS. These results may provide clues for biomarker screening for the clinical application of PARP inhibitors.

## CONFLICT OF INTEREST

All authors have declared that no potential conflicts of interest exist.

## AUTHOR CONTRIBUTIONS

QL and FLZ performed all the experiments and analyzed the data. QL wrote the manuscript. DYL provided some expression vectors and reagents. ZMS and DQL supervised the project.

## Data Availability

The data supporting the findings of this study are available from the corresponding author upon reasonable request.
