# Supplementary material for: USP9X stabilizes BRCA1 and confers resistance to DNA‐damaging agents in human cancer cells
Source: Cancer Med. 2019 Sep 11;8(15):6730–40. doi: 10.1002/cam4.2528 (PMC6825982; doi:10.1002/cam4.2528)
Supplement: Supplementary file 1 [file CAM4-8-6730-s001.pdf]

Supplementary information for

Lu et al. USP9X stabilizes BRCA1 and confers resistance to PARP inhibitor Olaparib in human cancer cells

Table S1. The shRNA and siRNA targeting sequences for the indicated genes

Genes	Sequences (5’-3’)
shUSP9X#1	5’-CGACCCTAAACGTAGACATTA-3’
shUSP9X#2	5’-CGATTCTTCAAAGCTGTGAAT-3’
siUSP9X#1	5’-AGAAAUCGCUGGUAUAAAUUU-3’
siUSP9X#2	5’-GCAGUGAGUGGCUGGAAGUTT-3’
siUSP9X#3	5’-GGACUUCUUUGAAAGUAAUTT-3’

Table S2 QPCR primers used in the study

Genes	Direction	Sequences (5′-3′)
USP9X	Forward	5′-AAGTGAAGCATGTCAGCGATT-3′
	Reverse	5′-GCCACACATAGCTCCACCA-3′
BRCA1	Forward	5′-TTGTTACAAATCACCCCTCAAGG-3′
	Reverse	5′-CCCTGATACTTTTCTGGATGCC-3′
GAPDH	Forward	5′-CGAGATCCCTCCAAAATCAA-3′
	Reverse	5′-TTCACACCCATGACGAACAT-3′

Table S3. The primary antibodies used in this study

Antibodies	Vendors	Cat#	Species	WB	IP
BRCA1	Santa Cruz	sc-6954	Mouse monoclonal	√	√
USP9X	CST	14898S	Rabbit monoclonal	√	√
V5	CST	13202S	Rabbit monoclonal	√	√
HA	CST	3724A	Rabbit monoclonal	√	√
Flag	Sigma	F1804	Mouse monoclonal	√	√
Vinculin	Sigma	V9131	Mouse monoclonal	√	
